# Documenting the Welfare and Role of Working Equids in Rural Communities of Portugal and Spain

**DOI:** 10.3390/ani10050790

**Published:** 2020-05-02

**Authors:** Emily Haddy, Joao B. Rodrigues, Zoe Raw, Faith Burden, Leanne Proops

**Affiliations:** 1 Centre for Comparative and Evolutionary Psychology, Department of Psychology, University of Portsmouth, Portsmouth PO1 2DY, UK; leanne.proops@port.ac.uk; 2 The Donkey Sanctuary, Sidmouth, Devon EX10 0NU, UK; joao.rodrigues@thedonkeysanctuary.org.uk (J.B.R.); zoe.raw@thedonkeysanctuary.org.uk (Z.R.); faith.burden@thedonkeysanctuary.org.uk (F.B.)

**Keywords:** animal welfare, donkey, EARS tool, equid welfare, *Equus asinus*, mule, welfare assessment, working equid

## Abstract

**Simple Summary:**

Understanding the social and cultural context of the role that working animals fulfil is crucial to improving their welfare. This study aimed to provide insight into the welfare status and traditional use of working equids in rural Western European communities using a new protocol for assessing working equid welfare, designed to provide a broad, holistic view of the welfare of working equids and the context in which they are found. Other questions on the topics of equid management practices, social transmission of expertise, environmental stressors, and traditions, alongside physical and behavioural welfare assessments were also included to explore the impact of these wide-ranging factors on an understudied population of working equids. A total of 60 working equid owners from rural communities in Portugal and Spain participated. Many owners stated that the help donkeys provided was invaluable, and donkeys were considered to be important for both farming and daily life. However, participants also recognised that the traditional agricultural way of life was dying out. Questions investigating the social transfer of information within the villages were effective in finding local sources of equid knowledge. Overall, welfare was deemed fair, and the protocol enabled the identification of the most prevalent welfare problems within the communities studied. The findings suggest that the new protocol is feasible, providing insights into the traditional practices, community structure, and beliefs of equid owners. Increasing understanding of the cultural context, social structure, and attitudes within a community may, in the future, help to make equid welfare initiatives more effective.

**Abstract:**

Recently, the need for a more holistic approach to welfare assessment has been highlighted. This is particularly pertinent in the case of working equids who provide vital support for human livelihoods, often in low- to middle-income countries, yet suffer from globally low standards of welfare. This study aimed to provide insight into the welfare status and traditional use of working equids in rural Western European communities using the new EARS welfare tool, designed to provide a broad view of the welfare of working equids and the context in which they are found. Other questions on the topics of equid management practices, social transmission of expertise, environmental stressors, and traditions, alongside physical and behavioural welfare assessments were also included to explore the impact of these wide-ranging factors on an understudied population of working equids. The protocol was trialled on 60 working equid owners from communities in Portugal and Spain where, despite the decline in traditional agricultural practices and livestock keeping, donkeys and mules remain working animals. Many owners stated that the help donkeys provided was invaluable, and donkeys were considered to be important for both farming and daily life. However, participants also recognised that the traditional agricultural way of life was dying out, providing insights into the traditional practices, community structure, and beliefs of equid owners. Questions investigating the social networks and social transfer of information within the villages were effective in finding local sources of equid knowledge. Overall, welfare was deemed fair, and the protocol enabled the identification of the most prevalent welfare problems within the communities studied, in this case obesity and the use of harmful practices. The findings suggest that the new protocol was feasible and detail how contextual factors may influence equid welfare. Increasing understanding of the cultural context, social structure, and attitudes within a community, alongside more traditional investigations of working practices and animal management, may, in the future, help to make equid welfare initiatives more effective.

## 1. Introduction

Animal welfare is a multifaceted concept [1] influenced by a variety of factors. As a consequence, its assessment is a complex process that, in order to be successful, must take these factors into account [2]. However, in the past, the focus on biological functioning in welfare evaluations, while neglecting animals’ emotional state or consciousness, was commonly seen [3]. The questionnaires accompanying traditional working animal welfare assessments have also typically focused on identifying working and management practices, rather than exploring the social and cultural context in which the animal is found [4].

Resulting from the need for a more holistic approach to welfare, a recent consultation took place to define a One Welfare conceptual framework. Born from, and partially overlapping, the One Health Initiative, One Welfare places emphasis on the interconnectedness of animal welfare, human wellbeing, and the environment [5]. It serves as a platform for interdisciplinary collaboration to improve both human and animal welfare on an international scale. One Welfare’s holistic approach considers scientific, ethical, economic, religious, and cultural issues within its framework [6]. For working animals worldwide, understanding the social and cultural context of the role that they fulfil is key to improving their welfare. This is especially pertinent in the case of working equids (donkeys, horses, and mules), which are often overlooked in higher level policy and agricultural interventions [7]. Over 100 million working equids provide vital support for human livelihoods in the developing world [8]. They are relied on for everyday activities, providing access to healthcare, education, and basic necessities in some of the most marginalised communities worldwide [7]. In some cases, working equids are people’s only source of income. Despite the fact that they are often people’s most important asset, welfare standards globally are low. Common welfare problems include insect exposure, poor body condition, lameness, trauma, and dehydration [9].

Traditionally, methods of equid welfare assessment have focussed on physical welfare markers and only more recently incorporated behavioural indicators of welfare [10]. Physical markers such as the presence of wounds and lameness are visible manifestations of poor welfare and are widely used as a metric in equid welfare studies [11,12,13]. Body condition scoring is also considered to be an effective welfare metric and is a commonly utilised tool [9,14,15]. For example, studies have successfully demonstrated links between behavioural markers and welfare problems: apathy in equids is associated with the presence of skin lesions, heat stress, poor body condition, and chronic pain [11,16], leading to the recommendation that communities with large numbers of apathetic animals should be considered high priority areas for welfare interventions [11]. Although initial concerns were raised regarding the subjectivity of behavioural markers of welfare, it has been shown that the measures are consistent and robust [17,18]. Despite these studies, there remains a lack of research into the development of holistic assessments that incorporate social science and human wellbeing, factors that are inextricably tied to animal welfare. 

Insights provided by local perspectives have been highlighted as crucial in the success of international development projects (which rely on how well social factors such as cultural norms, ethnic variations, and economic pressures are addressed) [19]. As such, qualitative methods are increasingly being employed as a tool in the field of international development to gather authentic contextual data. Understanding social networks of information is also crucial in ensuring the success of community participation programmes [20]. Research carried out on the implementation of equid welfare initiatives has highlighted the importance of community engagement and participation in order for the initiative to be successful [21]. Equid management and welfare practices are socially transferred information with owners learning from a wide range of sources. Community structure can affect how this information is transferred, with particular individuals influential in the potential acceptance of new practices [22,23]. However, most research carried out on working equids has focussed on direct indicators of equid welfare and working practices. As such, the social transfer of this information is an area that has, to date, received relatively little research attention. Some studies have investigated the most effective methods of transferring welfare information in an educational capacity [23]; however, there have been no investigations into the social networks that carry information regarding welfare practices within communities. Taking into account the social transfer of information within communities has the potential to make initiatives to improve equid welfare more effective. 

Assessments of working equine welfare must therefore consider the cultural context and the nature of the communities in which they are undertaken. Typically, studies of working equids are conducted in communities within low- to middle-income countries, and animals working in high-income countries, such as those found in Western Europe, are rarely considered. In the past century, the role of equids in Europe has changed significantly. Sports now represents the main economic purpose of equids in Europe, with the equine sector estimated to be worth 100 billion euros a year to the European economy [24]. Previous reliance on draught animals, including equids, for work has been replaced by motorisation and technological increases in many areas of the world [25]. However, rural communities using working equids still exist in many countries. In a report by World Horse Welfare and the Eurogroup for Animals [24], working equids were described as being used for agriculture including ploughing, working in the forestry industry, harnessing and transport of people and goods by cart, as well as those in the leisure industry. Working equids remain especially useful in areas where geographic constraints restrict the use of machinery for agriculture such as mountainous or steep land with difficult access. The decrease in the number of people living in these marginal, hilly areas, and hence the decrease in animals raised, was highlighted by Miraglia et al. [26] as having negative environmental impacts for the area including soil erosion, desertification, and forest fires. The welfare pressures facing the working equids of Europe are different from those identified for working equids in tropical developing countries, including environmental pressures and subsequent health issues such as skin problems [9,27]. As such, welfare assessments need to be able to account for the broad range of circumstances that equids encounter in their working lives. 

The aims of this study are twofold: first is to gain information on the types of welfare problems faced by working equids in Spain and Portugal, providing insights into the traditional way of life for these understudied European communities. Second is to test the feasibility of using the new EARS tool and structured interviews with working equid owners alongside assessment by researchers in these communities to incorporate the topics of the social transfer of knowledge and traditional practices alongside more standard measures of equid welfare.

## 2. Materials and Methods

### 2.1. Structured Interview and Assessment of the Animal

Data collection consisted of two parts, a welfare assessment of the animal, undertaken by the two first authors E.H. and J.B.R. (an equid veterinarian) and a structured interview with the equid owner. The welfare assessment protocol was taken from the new Equid Assessment Research and Scoping (EARS) tool developed by The Donkey Sanctuary (for more details, see [28]). This is the first welfare assessment tool developed for donkeys, horses, and mules and is designed to allow the standardised collection of data across the diverse contexts in which equids are found. The EARS tool is organised according to 19 welfare indicators, or subsections, that have previously been identified and recognised as having a substantial influence on welfare, such as housing, working conditions, end of life, transport, and health status. The full tool includes around 300 questions, the vast majority of them accompanied by a predefined list of optional answers, providing an extended series of questions designed to measure equid welfare in any context. The EARS tool allows the development of specific protocols for particular contexts and can be created according to the conditions in which the equid is kept, the research or management question, or the specific aims of the assessment. For example, the subset of questions relating to working conditions and housing are not relevant when assessing a feral population. For this specific study, the subsections relating to body condition, skin system, musculoskeletal system, behaviour, and health status were used for the welfare assessment of the animals. 

The structured interview was comprised of two different sets of questions. The first set was taken from the EARS tool and consisted of mainly quantitative questions focused on identifying key husbandry and working practices. A second set of qualitative open-ended questions, used to gather data on the social transfer of welfare knowledge regarding equid welfare practices and the impact of environmental challenge on working equids, was developed specifically for the purpose of this study. The relevant questions in the subsections relating to demographic data, housing, and working conditions were included in the structured interview. The subsection on housing contained assessment of the appropriateness of the housing, the cleanliness of the bedding, and access to water and was completed by the researchers (and omitted if the housing could not be viewed); all other questions were answered by the owner. The suitability of the housing was determined according to established U.K. government guidelines [29]. The second set of questions developed specifically for this study included questions on the topics of shelter, insect harassment, the social transfer of welfare knowledge, and livelihood factors. Prior to field-testing, the questions were reviewed by a panel of experts from The Donkey Sanctuary and the University of Portsmouth. The panel’s experience included field research in the intended pilot countries and experience of questionnaire production including production of the initial EARS tool. As a result, a number of amendments were completed before field-testing began, and these included re-wording a number of questions and adding the ability to choose multiple answers to some questions. See the Supplementary Materials (Tables S1 and S2) for a full list of the indicators used in the assessment of the animal.

### 2.2. Study Population

The participants interviewed were from rural villages, situated in the border region between the Northeast of Portugal and the Zamora Province in Spain, with data collected on both sides of the border. Participants were selected from key villages where it was known that working equids were used. Participants were selected on the basis of them owning and using working equids at the time of this study and their willingness to participate in the study. In the majority of cases (85%), interviews and examinations took place at the location where the equid was kept. This allowed evaluation of the condition of the equid’s housing. In a small number of cases, participants brought their animals to a central location such as the village square, and interviews and examinations took place at this location. In these cases (15%), it was not possible to view the equid’s housing arrangements.

A total of 60 equid owners participated in the study (women = 28, men = 32) with ages ranging between 47 and 91 years old (mean 71.3, SD = 9.5 years). Of the participants interviewed, ninety-five percent (*n* = 57) were farmers involved in small-scale agriculture. Equids were used for ploughing land, sowing, and harvesting of potatoes. Many of the participants were subsistence farmers, with the vegetables grown feeding them throughout the year. Some participants owned vineyards or grew olives for olive oil production as a main source of income; others farmed cattle as their main income source and used the equids to work with the cattle. Equids were ridden by owners to and from the location where the cattle were kept, used for transporting food for the cattle and for leaving with the cattle whilst they were grazing. Direct quotes referred to (presented as P followed by the participant number) resulted from owner discussion generated by any question during the interview. Many participants reported the reason for initially owning equids to be either working with cattle or to use equids as a replacement working animal when they sold their cattle (P42: “…when we sold the cows we changed and kept donkeys instead.”). Participants owning the traditional Mirandese and Zamorano-Leones breeds also received a European Union farming subsidy for maintaining native breeds [30]. Many owners mentioned this grant, some as a main reason for owning their donkeys, and some also bred foals for sale (P7: “Lots of people only have donkeys now because of the European subsidies”.

### 2.3. Subject Animals

A total of 57 donkeys (females = 52, stallions = 1, geldings = 4) and three mules (females = 1, geldings = 2) were assessed (these subject animals are latterly referred to collectively as working equids, although no horses were present in the sample). The primary roles of the equids assessed were as follows: 56.7% (*n* = 34) agroforestry, 21.7% (*n* = 13) breeding, 13.3% (*n* = 8) private riding, 5% (*n* = 3) transport of goods by cart, 3.3% (*n* = 2) pet. The average age of the equids assessed was 11.3 years (min = 3, max = 30, SD = 6.3 years).

### 2.4. Procedure

Working equid owners were interviewed by E.H. and J.B.R. (who is a fluent native speaker). The study was explained to potential participants, and informed consent was obtained, verbally rather than in writing, due to the participants’ level of literacy. For owners who had multiple equids, one equid per owner was selected at random to take part in the assessment. The owner was first asked to hold his/her equid while a short behavioural and physical welfare assessment was carried out (see Supplementary Materials Tables S1 and S2). The welfare metrics chosen were designed to take under 5 min to complete [31], making the examinations more practically feasible. Welfare markers included reaction to observer approach, signs of harmful practices, body condition score (on a 5 point scale, with 3 being ideal), presence of ectoparasites, skin alterations, and lameness. Cumulative analysis of all of these parameters resulted in a general health status rating of good, fair, or poor. Additional information regarding the suitability of housing was added by the researchers. Subsequently, owners were verbally asked a series of structured interview questions that covered topics including their working and welfare practices, the social transmission of knowledge regarding equid handling and welfare, protection from the elements, and livelihood factors. Interviews lasted on average between 15 and 30 min and were audio-recorded (with consent) for verification and subsequent transcription of qualitative data. Quantitative data were collected using a Samsung Galaxy tablet with Open Data Kit (ODK) Collect, a free, open-source application for Android devices, used by The Donkey Sanctuary for questionnaire design [32]. The research was approved by the University of Southampton’s Ethics Committee (ID: 31814) and adhered to the EU Directive 2019/63/EU for animal experiments and the Association of Animal Behaviour guidelines for the treatment of animals. All owners gave informed consent for both their participation and their animal’s inclusion in the study.

### 2.5. Data Analysis

Responses to open-ended questions were transcribed and content analysis used to identify common themes and consensus relating to practices and management of equids. These included contextual or cultural factors that may influence welfare, with the coding categories derived from the data [33]. Chi-squared tests (3 × 2 and 2 × 2) were used to test for differences in relevant welfare markers based on the owner’s perspectives and welfare practices. Owner practices tested as independent variables included: use of practices against insects and use of harmful practices. For analysis of the reaction to observer approach, the categories “moved head away from assessor”, “moved whole body away from assessor”, and “showed aggressive behaviour” were grouped together as negative reactions; these were analysed against the positive reaction “friendly approach” and the neutral reaction “did not move”. Analyses were performed using SPSS Version 24.0 (IBM Corporation, New York, NY, USA) [34]. 

## 3. Results

### 3.1. Quantitative Results

#### 3.1.1. Welfare Assessment

Physical assessment: Signs of harmful practices were observed in 60% (*n* = 36) of equids; 55% (*n* = 33) showed signs of limb tethering or hobbling; and 6.6% (*n* = 4) showed signs of the use of serreta or similar (abrasive metallic pieces used in the nose band or chin strap regions). Skin system alterations were observed in 80% (*n* = 48) of equids assessed: 51.8% (*n* = 31) had scars; 33.5% (*n* = 20) of equids had open wounds (mainly due to cutaneous habronemiasis and superficial injuries most often located on the legs); 31.7% (*n* = 19) had alopecia; and 5% (*n* = 3) had swellings. In the body condition score assessment (scored on a 1–5 scale), eight-point-three percent (*n* = 5) were (2) thin/moderate, 28.3% (*n* = 17) (3) ideal, 35% (*n* = 21) (4) fat, and 28.3% (*n* = 17) (5) very fat/obese (mean body condition score = 3.8 ± 0.9). Signs of illness recorded included presence of eye discharge 50% (n = 30), nasal discharge 8.5% (*n* = 5), unhealthy coat 5% (*n* = 3), signs of diarrhoea 3.3% (*n* = 2), quidding 1.7% (*n* = 1), and a clouded eye 1.7% (*n* = 1). Signs of heat stress were observed in 3.3% (*n* = 2) of equids, and 87% (*n* = 54) showed indicators of the presence of endo-/ecto-parasites. Of the equids assessed, 23.3% (*n* = 14) were lame, with three animals unable to be assessed for lameness due to situational constraints. The incidence of lameness showed a trend towards being higher when owners used harmful practices in comparison to when they did not, χ^2^ = (1, N = 57) = 3.26, *p* = 0.07. Of the equids assessed, the general health status (cumulatively scored based on the welfare parameters assessed) was deemed to be good in 26.7% (*n* = 16) of the animals, fair in 65% (*n* = 39) of the animals, and poor in 8.3% (*n* = 5) of the animals.

Behavioural assessment: At a distance, the general attitude of 88% (*n* = 53) of equids was alert and 11.7% (*n* = 7) was relaxed. The number of behavioural signs of insect nuisance in 1 min ranged from 0 to 74 (mean 11.4 ± 14.7). In response to the assessor’s approach, 53.3% (*n* = 32) of equids showed a friendly approach, 30% (*n* = 18) did not move, 13.3% (n = 8) moved their head away from the assessor, 1.7% (*n* = 1) moved their whole body away from the assessor, and 1.7% (*n* = 1) showed aggressive behaviour. Chin contact was accepted in 85% (*n* = 51) of cases, with 15% (*n* = 9) avoiding chin contact. In response to the assessor walking down the side of the equid, 46.7% (*n* = 28) showed a positive reaction, 48.3% (*n* = 29) a neutral reaction, and 5% (*n* = 3) a negative reaction. Tail tuck behaviour was displayed in 5% (*n* = 3) of cases and other signs of fear and distress in 3.3% (*n* = 2) of cases. There was no significant difference in equid reaction to observer approach between those whose owner used harmful practices and those whose owner did not, χ^2^ = (2, N = 60) = 1.17, *p* = 0.56. Owner behaviour in relation to the equid was recorded at the beginning of the interview process when the owner was in direct contact with the equid. All owners were scored as relaxed and confident. 

#### 3.1.2. Management Practices

The majority of equids (87%, (*n* = 54)) worked less than five days a week, and 81% (*n* = 49) worked for 3 h or less per day. Access to shelter after the working period was provided for 81.7% (*n* = 49) of equids assessed, and 13.3% (*n* = 8) had access to natural shade. In 44% (*n* = 27) of cases, the dimensions of the stable or shelter provided for the equid were not satisfactory [29], and in 75% (*n* = 45) of cases, access to water was limited. In 8% (*n* = 5) of cases, no bedding material was provided; in 23% (*n* = 14) of cases, bedding was insufficient; and in 58% (*n* = 36) of cases, bedding was dirty. Pasture and hay were the main dietary components with only 5% (*n* = 3) of equids not fed pasture and 13.3% (*n* = 8) not fed hay. Owners stated that during this season, their animals could graze freely all day and night in 80% (*n* = 48) of cases and all night in 13.3% (*n* = 8) of cases; food was provided 2–3 times daily in 1.7% (*n* = 1) of cases and 1–2 times daily in 5% (*n* = 3) of cases. The majority of owners (63.3% (*n* = 38)) said that they did try to stop insects from biting their equid. Of those owners who tried to stop insects from biting their equid, 13 reported the use of a lotion or repellent, including chemical antiparasitic products. Avoiding areas of high insect density such as moving their equid into the stable at peak fly times was reported by five owners. The most common practice, reported by 16 owners, was the use of oil as a repellent (often applied with a chicken feather). Mainly, this was olive oil; although two used burned oil from the tractor, and one applied butter. There was no significant difference in the presence of ectoparasite indicators between equids whose owners used practices to stop insects from biting them and those who did not, χ^2^ *=* (1, N = 58) = 0.004, *p =* 0.95. 

#### 3.1.3. Social Networks

All owners were experienced with equids: 85% (*n* = 51) of owners stated that they had worked with equids for all of their working life with the other 15% (*n* = 9) having worked with equids for more than five years. Virtually all owners, 95% (*n* = 57), learned about their equid’s daily needs and handling from traditional family knowledge; 1.7% (*n* = 1) learned through an employer; and 3.3% (*n* = 2) taught themselves. Questions investigating the social networks and social transfer of information within the villages were effective in finding local sources of equid knowledge. The question, “Is there a particular person in your community who you think is good with equids?” was successful in identifying key individuals within a village. These individuals were people that others would ask for advice on equid management, especially if problems were encountered with their own animals. These key individuals were perceived to be particularly good for two reasons: most frequently because they had much experience with equids, but also because they owned many animals. Within villages, these data were triangulated because multiple participants confirmed the same key individual. Within one village, the questions identified a key individual from the past, now deceased, with participants commenting that “now there is no-one to ask” (P52). One participant revealed that her position within the social network of equid owners in the village was inherited (P51: “The fact that my father was the guy with the stallion that serviced all of the females, that was important and respected. I have kept that status because I am his daughter.”). In the case of one village, a local shop that sold medicines served as a point of advice; however, within some villages, there was no key individual identified by any members of the community (P29: “…everyone single person has an opinion on what you should do but in the end there is no-one I would ask.”). The information gathered provided an understanding of the context and drivers of social networks within communities and how these may vary from village to village. Given the high average participant age and the fact that the vast majority of participants learned their management practices from family or traditional knowledge, this indicated that equid management practices were unlikely to have changed in a long period of time. Almost all (91.7% (*n* = 55)) owners said that they liked working with equids, demonstrating the importance of the relationship between owners and their animals.

### 3.2. Qualitative Results

The two main themes identified though qualitative analysis of the open questions were the importance and utility of donkeys for people’s everyday life and the acknowledgement that their traditional way of life was coming to an end. These themes provided contextual background required in order to understand the prevailing influences upon equid welfare within the sample community.

#### 3.2.1. Utility to People

Comments relating to the animals’ utility to people were expressed mainly in response to the questions “What is the primary role of the equid?” and “What does your working equid mean to you?” Motorisation is increasingly replacing traditional agricultural methods such as ploughing using animals. Tractors can more rapidly cover larger areas with less physical effort for farmers in comparison to labour-intensive traditional methods. Many participants commented that they used to plough using cows, but now use tractors for the majority of their work (P13: “Work was done with cows, then the tractor arrived, and donkeys stayed for the light work”). Some of the land used for growing vegetables was unsuitable for the tractor due to its steepness or small size; therefore, equids were still used to plough these areas (P34: “There are things I cannot make with a tractor because they are too big.”). Cattle prominently featured when asking participants about the social status associated with owning equids, with many stating that previously, it had been the cows that had been associated with higher status, and those who could not work with cows worked with donkeys (P30: “I started because I didn’t have the chance to have cows so I had to work with donkeys.”). When asked which species of equid was most beneficial to own, many replied that cows were better for working than any equid species (P32: “Those who say good things about donkeys have never ploughed with cows.”). However, despite the sale of the majority of other livestock, equids have remained an integral part of life within these communities, and this was partially due to their versatility. Equids were used for a variety of roles including: riding (P30: “You have your car, for me this is my car!”), pulling goods in a cart including animal feeds, manure, vegetables, fire wood, and vines, and ploughing (P33: “We plough the pumpkins then we rest for 15 min in between. After we plough the potatoes”). A theme that appeared strongly in the qualitative analysis was the utility of equids to people and their reliance on this help. It was clear that equids were still considered to be important for both farming and daily life, and to some indispensable (P54: “More than social status it is something necessary, especially in the old days for daily work, if you didn’t have a donkey you couldn’t do many things.”, P13: “…old people give them high value because they need them”). One participant did not know how they would work without the help of their equid (P46: “She helps a lot. If it was not for the animal what would I do?”), and those who could not afford access to other methods carried out all farming work with equids (P48: “We don’t have a tractor so we do everything with the animals.”). Participants described their regular use of equids for “daily tasks” and “light work”. Women in particular seemed to value the help that equids provided, with one participant highlighting that her husband worked using machinery, but that she worked with the equids (P35: “If my husband is going to collect the wood he goes with the tractor, but I go with the cart.”).

Participants acknowledged their advanced age (P29: “She was very lively when she was young, it was difficult to put her to work. Now she is as old as me!”, P4: “I like working with donkeys but I’m getting old”), with many experiencing heath issues and limited mobility; however, the calm nature of donkeys in comparison to other equids made it possible for older participants to handle and keep them. The Mirandese and Zamorano-Leones breeds in particular are known for their relaxed and friendly temperaments [35]. This was reflected in the comments made by participants (P42: “Cows were too much for me, donkeys are calmer”, P9: “I had a mule before and I preferred it but now that I’m older I have a donkey”). When participants were asked to describe the personality of their equid, the majority of answers given were positive, with 25 responses containing the word “good” to describe the equid. “Calm”, “friendly”, and “docile” were also common answers, mentioned 12, 10, and three times, respectively. This was a reflection of the qualities that the Mirandese and Zamorano-Leones donkeys are renowned for, qualities that were particularly valued by the participants interviewed. Other adjectives used included: “sweet”, “honest”, “intelligent”, “humble”, “strong”, and “brave”. The negative descriptions given primarily related to the equids not being docile. One participant stated that when the donkey was young, she was lively and difficult to work with. Another described their equid as “stubborn”, and others mentioned “not calm” or “less calm than the old donkey”. Reducing their physical effort by working with donkeys allowed participants in their 80s and 90s to continue farming. One participant commented that their donkey gave them independence, meaning that they did not have to ask or depend on anyone else (P57: “Happiness of doing something that doesn’t depend on anyone else”). This could be especially important to older people, for whom loss of independence is associated with depression and institutionalisation [36,37]. 

#### 3.2.2. Traditional Way of Life

The recognition that the traditional way of life was soon coming to an end featured prominently throughout the qualitative analysis; the question “Within your society do you think owning an equid affects your status?” often elicited discussion about the changes in farming and comparison between “old times” and the present. The majority of participants were elderly (mean 71.3 years, SD 9.5), and 85% had worked with equids for their entire lives. However, the number of traditional farmers has rapidly decreased over time, with younger generations increasingly moving into urban areas and pursuing alternative careers [35]. Without people to take over the farming, a large amount of land is now abandoned with mixed forest increasing by 91% between 1990 and 2005, replacing fields previously farmed for cereals in the study area of Portugal [38]. Many participants commented on the younger generation’s lack of interest in and understanding of traditional agricultural life (P58: “For the older generation it is important but not for the young people, they don’t care.”).

It was clear from the comments made by participants that over their lifetimes, they had seen a large reduction in traditional farming and the number of animals kept in the villages (P20: “…only our 3 and 1 more [donkey] left in a village that used to have over 100”). Due to the decline in traditional farming using equids, the status that was once attached to owning equids or cattle no longer remained (P6: “…old time was cows high status, donkey low, but now no one has cows and it doesn’t matter.”). Some participants commented that this status has now been transferred instead to the ownership of tractors (P3: “…donkeys don’t affect status now. Used to be cattle was high status and donkeys less but now it is tractors.”); however, others now thought that, in light of the imminent extinction of the traditional lifestyle, it no longer mattered (P29: “Everything is going to end soon so social status is not really a concern!”, P56: “It doesn’t matter, it is history.”). 

## 4. Discussion

Questions from this new welfare assessment protocol were received well by participants; there were no questions that elicited refusals to answer or adverse reactions, and the general perception of the interview process was positive. Interviews were pre-arranged and adapted to fit the routine of participants in order to minimise disruption to their daily lives. One participant who had not been contacted for interview heard about the data collection by word of mouth and volunteered to be interviewed. Field testing allowed the length of the interview to be established. The length of the assessment was felt to be an appropriate balance between the detail of answers gained and the speed of assessment. For owners taking time out of their working day to complete the interview, an excessive length could result in reluctance to participate. The process lasted between 15 and 35 min in total. In general, owners seemed happy with the length of time taken to complete the questions with only a couple of participants asking how much longer the process would take. It was found that a routine in which one person assessed the equid and the other recorded the answers was optimally efficient for the behavioural and physical assessments, although the process could be completed by one researcher alone. After the physical and behavioural assessments were completed, it was not necessary to have the equid present for the remainder of the protocol. Owners frequently re-stabled the equids before answering further questions; this prevented the need for the equid to be restrained for longer than necessary and proved easier for owners. 

The use of the welfare assessment section of the EARS Tool was successful in identifying differences in the physical and behavioural welfare indicators across equids observed and enabled us to identify key welfare issues. Weight was identified as one of the most prevalent welfare problems with over half of the equids assessed as overweight. This is unusual in a population of working equids, being more commonly seen in companion and pet equids [39,40]; however, most equids in the sample worked less than five days a week and for three hours or less per day. Therefore, weight problems could be largely due to the seasonal nature of agricultural work, leaving the equids without a high level of physical exercise for long periods of time despite still being fed on good quality forage. The forage observed in nearly all cases was home-grown oat hay, high in energy as it is harvested when the grain is still soft and is not completely formed. Overweight equids are at higher risk of suffering from health conditions including laminitis and hyperlipemia [41] and as such present a welfare challenge. 

The use of harmful practices was also highlighted as a welfare issue, and two types were recorded: hobbling/leg tethering and the use of serreta, with hobbling/leg tethering the most commonly observed harmful practice. The majority were hobbled or tethered whilst turned outside to prevent them from escaping due to a lack of effective field boundaries. One participant hobbled their donkeys in order to keep them close to the cows, and two reported only hobbling their donkeys when they were in oestrus. Lameness in equids whose owners used harmful practices showed a trend towards being higher in comparison to owners who did not. The hobbles observed during the study were metal chains, applied by the owners distal to the carpal/tarsal area of the animals. Hobbling can cause painful leg injuries including lesions, infections, and swelling, especially in the pastern region where the chains are tied to the animal [42]. Damage and inflammation of soft tissue can lead to lameness, and hobbling of working equids has been reported to cause complications leading to loss of working days [43]. No difference was found between reaction to observer approach in equids whose owners used harmful practices and those whose owners did not. This may be due to the low frequency of use of harmful practices; some owners reported only hobbling the equids temporarily or for a particular reason. Fearful or aggressive reactions to observer approach may also be associated more with general handling practices and interaction between owner and equid; it has been demonstrated that inappropriate handling can elicit fear of humans [44]. All owners appeared relaxed and confident with their animals. 

There was no statistically significant difference in indicators of ectoparasite presence between owners that tried to stop insects from biting their equids and those who did not. This suggests that practices used may not be effective against the types of flies observed (which included flies from the genus *Tabanus* and *Hippobosca*). Motor oil has been shown to reduce the attraction of house flies to a honey solution [45], and olive oil has been demonstrated to be a feed repellent against the mosquito species *C. quinquefasciatus* [46], providing support for the traditional practices shown; however, neither has been demonstrated to be an effective ectoparasite repellent in equids. Burned oil (as well as other caustic products) are often also used on open wounds, which is problematic as it delays the healing process [47]. Antiparasitics are usually targeted at specific parasites such as worms and will have no effect on flies, although specific ectopic products are available for use against lice and flies. The data in this study did not distinguish between these two types of products. 

## 5. Conclusions

For these communities in Spain and Portugal, the main welfare concerns were weight, the use of harmful practices, and the presence of scars and superficial wounds. The qualitative results highlighted the continued importance of these animals to a community where many are concerned that the traditional way of life is ending and social networks of knowledge transfer are being broken. The results demonstrated that the EARS tool was an effective way to identify prevalent welfare issues within a community, allowing for prioritisation in any future welfare initiatives. The inclusion of a wide range of open and closed questions that covered management questions, welfare assessment, cultural practices, and owner attitudes allowed for a more holistic appraisal, not just of the current welfare state of the working equids studied, but also the contextual background that was required in order to understand the prevailing influences on equid welfare within the sample community. In the future, the protocol could potentially provide a valuable insight into the social transfer of knowledge within working equid owning communities and will help to identify key individuals who are influential within traditional networks of social knowledge, making welfare programs more effective. The protocol could also inform understanding of the traditional practices, social structure, and beliefs of the people within the community being studied. 

## Supplementary Materials

The following are available online at https://www.mdpi.com/2076-2615/10/5/790/s1, Table S1: Behavioural Assessment Criteria; Table S2: Physical Assessment Criteria.

## References

[1] Fraser D. (1995). Science, Values and Animal Welfare: Exploring the “Inextricable Connection”. Anim. Welf..

[2] Mimms S., May E., Butterworth A. (2007). Definition of criteria for overall assessment of animal welfare. Anim. Welf..

[3] Rushen J. (2003). Changing concepts of farm animal welfare: Bridging the gap between applied and basic research. Appl. Anim. Behav. Sci..

[4] Luna D., Vásquez R., Rojas M., Tadich T. (2017). Welfare Status of Working Horses and Owners′ Perceptions of Their Animals. Animals.

[5] One Welfare One Welfare Initiative. https://www.onewelfareworld.org/about.html.

[6] Pinillos R.G., Appleby M., Manteca X., Scott-Park F., Smith C., Verlade A. (2016). One Welfare―A platform for improving human and animal welfare. Vet. Rec..

[7] Stringer A. (2014). Improving animal health for poverty alleviation and sustainable livelihoods. Vet. Rec..

[8] FAOSTAT FAO Statistical Year Book. Food and Agricultural Organisation of the United Nations. http://www.fao.org/faostat/en/#home.

[9] Sánchez-Casanova R.E., Masri-Daba M., Alonso-Díaz M.Á., Méndez-Bernal A., Hernández-Gil M., Fernando-Martínez J.A. (2014). Prevalence of cutaneous pathological conditions and factors associated with the presence of skin wounds in working equids in tropical regions of Veracruz, Mexico. Trop. Anim. Health Prod..

[10] Hockenhull J., Whay H.R. (2014). A review of approaches to assessing equine welfare. Equine Vet. Educ..

[11] Burn C.C., Dennison T.L., Whay H.R. (2010). Relationships between behaviour and health in working horses, donkeys, and mules in developing countries. Appl. Anim. Behav. Sci..

[12] Reix Nèe Broster C.E., Burn C.C., Pritchard J.C., Barr A.R.S., Whay H.R. (2014). The range and prevalence of clinical signs and conformation associated with lameness in working draught donkeys in Pakistan. Equine Vet. J..

[13] Whay H.R., Dikshit A.K., Hockenhull J., Parker R.M.A., Banerjee A., Hughes S.I., Pritchard J.C., Reix C.E. (2015). Evaluation of changes in equine care and limb-related abnormalities in working horses in Jaipur, India, as part of a two year participatory intervention study. PLoS ONE.

[14] Pritchard J.C., Lindberg A.C., Main D.C.J., Whay H.R. (2005). Assessment of the welfare of working horses, mules and donkeys, using health and behaviour parameters. Prev. Vet. Med..

[15] Usman S., Disassa H., Kabeta T., Zenebe T., Kebede G. (2015). Health and Welfare Related Assessment of Working Equine in and Around Batu Town, East Shoa, Central Ethiopia. Nat. Sci..

[16] Swann W.J. (2006). Improving the welfare of working equine animals in developing countries. Appl. Anim. Behav. Sci..

[17] Regan F.H., Hockenhull J., Pritchard J.C., Waterman-Pearson A.E., Whay H.R. (2014). Behavioural repertoire of working donkeys and consistency of behaviour over time, as a preliminary step towards identifying pain-related behaviours. PLoS ONE.

[18] Minero M., Dalla Costa E., Dai F., Murray L.A.M., Canali E., Wemelsfelder F. (2016). Use of Qualitative Behaviour Assessment as an indicator of welfare in donkeys. Appl. Anim. Behav. Sci..

[19] Mack N., Woodsong C., MacQueen K., Guest G., Namey E. (2005). Qualitative Research Methods: A Data Collector’s Field Guide.

[20] Brown J. (2014). Evaluating Participatory Initiatives in South Africa. SAGE Open.

[21] Upjohn M.M., Attwood G.A., Lerotholi T., Pfeiffer D.U., Verheyen K.L.P. (2013). Quantitative versus qualitative approaches: A comparison of two research methods applied to identification of key health issues for working horses in Lesotho. Prev. Vet. Med..

[22] Knight J., Weir S., Woldehanna T. (2003). The role of education in facilitating risk-taking and innovation in agriculture. J. Dev. Stud..

[23] Stringer A.P., Bell C.E., Christley R.M., Gebreab F., Tefera G., Reed K., Trawford A., Pinchbeck G.L. (2011). A cluster-randomised controlled trial to compare the effectiveness of different knowledge-transfer interventions for rural working equid users in Ethiopia. Prev. Vet. Med..

[24] World Horse Welfare and Eurogroup for Animals (2015). Removing the Blinkers: The Health and Welfare of European Equidae in 2015.

[25] Cerutti A.K., Calvo A., Bruun S. (2014). Comparison of the environmental performance of light mechanization and animal traction using a modular LCA approach. J. Clean. Prod..

[26] Miraglia N., Burger D., Kapron M., Flanagan J., Langlois B., Martin-Rosset W., Rubino R., Sepe L., Dimitriadou A., Gibon A. (2006). Local animal resources and products in sustainable development: Role and potential of equids. Livestock Farming Systems―Product Quality Based on Local Resources Leading to Improved Sustainability.

[27] Popescu S., Diugan E.A. (2013). The Relationship between Behavioral and Other Welfare Indicators of Working Horses. J. Equine Vet. Sci..

[28] Raw Z., Rodrigues J.B., Rickards K., Ryding J., Norris S.L., Judge A., Kubasiewicz L.M., Watson T.L., Little H., Hart B. (2020). Equid assessment, research and scoping (EARS): The development and implementation of a new equid welfare assessment and monitoring tool. Animals.

[29] DEFRA (2017). Code of Practice for the Welfare of Horses, Ponies, Donkeys and Their Hybrids.

[30] European Commission On-farm Conservation of Rare and Endangered Local Animal Breeds. http://ec.europa.eu/environment/nature/rbaps/fiche/-farm-conservation-rare-and-%0Aendangered-local-anima_en.htm.

[31] Leeb C., Henstridge C., Dewhurst K., Bazeley K. (2003). Welfare assessment of working donkeys: Assessment of the impact of an animal healthcare project in West Kenya. Anim. Welf..

[32] Hartung C., Lerer A., Anokwa Y., Tseng C., Brunette W., Borriello G. Open data kit: Tools to build information services for developing regions. Proceedings of the 4th ACM/IEEE International Conference on Information and Communication Technologies and Development (ICTD ’10).

[33] Hsieh H.-F., Shannon S.E. (2005). Three Approaches to Qualitative Content Analysis. Qual. Health Res..

[34] (2016). IBM Corporation IBM SPSS Statistics for Windows.

[35] Porter V., Alderson G.L.H., Hall S.J.G., Sponenberg D.P. (2016). Mason’s World Encyclopedia of Livestock Breeds and Breeding.

[36] Kennedy G.J., Kelman H.R., Thomas C. (1990). The emergence of depressive symptoms in late life: The importance of declining health and increasing disability. J. Community Health.

[37] Covinsky K.E., Palmer R.M., Fortinsky R.H., Counsell S.R., Stewart A.L., Kresevic D., Al E., Burant C.J., Landefeld C.S. (2003). Loss of independence in activities of daily living in older adults hospitalized with medical illness: Increased vulnerability with age. J. Am. Geriatr. Soc..

[38] Silva J.S., Vaz P., Moreira F., Catry F., Rego F.C. (2011). Wildfires as a major driver of landscape dynamics in three fire-prone areas of Portugal. Landsc. Urban Plan..

[39] Cox R., Burden F., Proudman C.J., Trawford A.F., Pinchbeck G.L. (2010). Demographics, management and health of donkeys in the UK. Vet. Rec..

[40] Dugdale A.H.A., Curtis G.C., Cripps P., Harris P.A., Argo C.M. (2010). Effect of dietary restriction on body condition, composition and welfare of overweight and obese pony mares. Equine Vet. J..

[41] Burden F., Thiemann A. (2015). Donkeys Are Different. J. Equine Vet. Sci..

[42] Brooke Hospital for Animals (2016). Welfare Interpretation Manual.

[43] Ahmad A., Zaman S.F., Aravindan M., Thanammal S.R. A study about the knowledge, attitudes and the practice of hobbling equids in Meerut district, India. Proceedings of the 6th International Colloquium on Working Equids: Learning from Others.

[44] Rushen J., Taylor A.A., Marie De Passille A. (1999). Domestic animals’ fear of humans and its effect on their welfare. Appl. Anim. Behav. Sci..

[45] Maganga M.E., Gries G., Cries R. (1996). Repellency of Various Oils and Pine Oil Constituents to House Flies (Diptera: Muscidae). Environ. Entomol..

[46] Amer A., Mehlhorn H. (2006). Repellency effect of forty-one essential oils against Aedes, Anopheles, and Culex mosquitoes. Parasitol. Res..

[47] Rodrigues J.B. (2020). Personal Communication.

