# Supplementary material for: Documenting the Welfare and Role of Working Equids in Rural Communities of Portugal and Spain
Source: Animals (Basel). 2020 May 2;10(5):790. doi: 10.3390/ani10050790 (PMC7277599; doi:10.3390/ani10050790)
Supplement: Supplementary file 1 [file animals-10-00790-s001.pdf]

Table S1. Behavioural Assessment Criteria.

**Indicator**	**Criteria**
General attitude of the equid at a distance.<br>Assessor observes the equid from a distance of 3-5 metres before approaching.	1) At ease – Relaxed, calm and/or resting.<br>2) Alert – Actively interested in surroundings. Responsive to environmental stimuli. Ears moving, eyes open, movement to keep away flies.<br>3) Apathetic – depressed and withdrawn. Lack of response to surroundings, head lowered, eyes may be closed, lack of movement to keep away flies.<br>4) Agitated – Aggressive and hyper-reactive to stimuli.
Response to observer approach<br>Assessor approaches the equid's head at an angle of 20° (rather than directly in front of the equid). Assessor stops 30cm from the equids head and records reaction at the time of stopping.	1) Friendly – head turns towards the assessor.<br>2) Does not move – no reaction to assessor presence.<br>3) Turns head away – moves head away from assessor.<br>4) Turns whole body away - moves entire body away from assessor<br>5) Aggressive – flattens ears, attempts to bite or kick assessor.
Response to walking down the side of the equid<br>Assessor walks alongside the equid without touching it, from the head to the rear at a distance of 30cm from the equid.	1) Positive – calm and stationary, ears rotate towards the assessor, maintains contact with assessor.<br>2) Neutral – No reaction to the movement of the assessor.<br>3) Negative – avoidance responses, ears flattened, attempts to flee, trying to bite or kick assessor.
Tail tuck<br>Assessor records whether equid (donkeys and mules only) shows a tail tuck response.	1) Absent – tail relaxed<br>2) Present – clamps or tucks tail in hindquarters
Head and neck position<br>Assessor records the position of the equid's head and neck.	1) Head above withers<br>2) Head level with withers<br>3) Head lower than withers
Ear position<br>Assessor records the position of the equid's ears.	1) Both ears forwards<br>2) Both ears facing sideways<br>3) Both ears backwards but not flattened<br>4) Both ears flattened towards neck<br>5) Combination – ears in different directions
Chin contact<br>Assessor raises a cupped hand towards the equid's chin and if contact is accepted, touches the chin.	1) Accepts chin contact<br>2) Avoids chin contact
Movement of the equid's tail	1) Hanging still

Assessor records tail movement	2) Swishing
Response to external stimuli<br>If assessor had observed response to environmental stimuli during assessment this was recorded, if not assessor clapped hands above head and recorded the equid's reaction.	1) Response to stimuli present<br>2) Response to stimuli absent
Signs of fear and distress present<br>Assessor recorded any other behavioural signs of distress or fear observed during the assessment.	1) None<br>2) Showing the whites of the eyes<br>3) Unpredictable or sudden movements<br>4) Sudden startle responses when standing quietly – rapidly lifting the head<br>5) Aggressive behaviour – ears flattened, attempts to bite and/or kick<br>6) Trembling<br>7) Head shyness – the equid avoids any contact with its head<br>8) Complete withdrawal
Behavioural signs of insect nuisance<br>Assessor counts the number of behavioural signs of insect nuisance during one minute	1) Number of behaviours observed – behaviours include head shaking, tail swishing, foot stamping & skin flickering.

Table S2. Physical Assessment Criteria

**Indicator**	**Criteria**
Harmful Practices<br>Assessor examines the equid for signs of harmful practices that may affect welfare	1) None<br>2) Signs of hot brand<br>3) Signs of firing<br>4) Signs of limb tethering or hobbling<br>5) Signs of amputations or mutilations including ears, tail and nostrils<br>6) Signs of the use of live serrata or metallic chains in the noseband<br>7) Other
Body Condition Score<br>Assessor examines the equid by observation and touch to assess body fat and condition. No half measures were included	1) Very thin/Poor – ribs, spine and hips prominent, pelvis hollow<br>2) Thin/Moderate – ribs, spine and hips visible, pelvis flat<br>3) Ideal – spine just visible, pelvis slightly rounded<br>4) Fat – spine not visible, pelvis rounded<br>5) Very fat/Obese – neck convex, pelvis rounded, 'gutter' along spine.
Indicators of the presence of endo or ecto-parasites<br>Assessor examines the equid's skin and coat for signs of parasites.	1) No indicators<br>2) Signs of endo-parasites<br>3) Signs of ectoparasites – flies<br>4) Signs of ecto-parasites – midges<br>5) Signs of ecto-parasites - lice<br>6) Signs of ecto-parasites - ticks<br>7) Signs of ecto-parasites - other
Skin System alterations	1) No alterations

Assessor examines the equid’s skin for signs of alterations.	2) Open wounds<br>3) Scars<br>4) Alopecia<br>5) Sarcoids<br>6) Swellings<br>7) Other
Lameness<br>Assessor asks the owner to walk their equid a few paces and observes the equid’s movement.	1) Severely lame – unable to walk or lying, unable to stand.<br>2) Lame but still moving<br>3) No lameness
Other signs of illness<br>Assessor notes other clinical signs of illness not previously recorded.	1) Nasal discharge<br>2) Eye discharge<br>3) Signs of diarrhoea<br>4) Unhealthy coat<br>5) Significant discharge from penis or vulva<br>6) Abdominal pain<br>7) Other
General Health Status<br>Taking into account observed health indicators assessor scores the general health of the equid.	1) Good<br>2) Fair<br>3) Poor
Thermal Stress<br>Assessor records any physical symptoms of thermal stress.	1) No indicators<br>2) Indicators of cold stress – high respiration, shivering, abnormally coloured mucous membranes.<br>3) Indicators of heat stress – sweating, high respiration, open nostrils.
